# Processing of pitch and location in human auditory cortex during visual and auditory tasks

**DOI:** 10.3389/fpsyg.2015.01678

**Published:** 2015-11-06

**Authors:** Suvi Häkkinen, Noora Ovaska, Teemu Rinne

**Affiliations:** ^1^Institute of Behavioural Sciences, University of HelsinkiHelsinki, Finland; ^2^Advanced Magnetic Imaging Centre, Aalto University School of ScienceEspoo, Finland

**Keywords:** auditory cortex, attention, human, pitch processing, spatial processing

## Abstract

The relationship between stimulus-dependent and task-dependent activations in human auditory cortex (AC) during pitch and location processing is not well understood. In the present functional magnetic resonance imaging study, we investigated the processing of task-irrelevant and task-relevant pitch and location during discrimination, n-back, and visual tasks. We tested three hypotheses: (1) According to prevailing auditory models, stimulus-dependent processing of pitch and location should be associated with enhanced activations in distinct areas of the anterior and posterior superior temporal gyrus (STG), respectively. (2) Based on our previous studies, task-dependent activation patterns during discrimination and n-back tasks should be similar when these tasks are performed on sounds varying in pitch or location. (3) Previous studies in humans and animals suggest that pitch and location tasks should enhance activations especially in those areas that also show activation enhancements associated with stimulus-dependent pitch and location processing, respectively. Consistent with our hypotheses, we found stimulus-dependent sensitivity to pitch and location in anterolateral STG and anterior planum temporale (PT), respectively, in line with the view that these features are processed in separate parallel pathways. Further, task-dependent activations during discrimination and n-back tasks were associated with enhanced activations in anterior/posterior STG and posterior STG/inferior parietal lobule (IPL) irrespective of stimulus features. However, direct comparisons between pitch and location tasks performed on identical sounds revealed no significant activation differences. These results suggest that activations during pitch and location tasks are not strongly affected by enhanced stimulus-dependent activations to pitch or location. We also found that activations in PT were strongly modulated by task requirements and that areas in the inferior parietal lobule (IPL) showed task-dependent activation modulations, but no systematic activations to pitch or location. Based on these results, we argue that activations during pitch and location tasks cannot be explained by enhanced stimulus-specific processing alone, but rather that activations in human AC depend in a complex manner on the requirements of the task at hand.

## Introduction

Prevailing auditory models suggest that human auditory cortex (AC) consists of anatomically and functionally separate fields that are organized into two or more segregated hierarchical processing streams (Rauschecker and Scott, 2009; McLachlan and Wilson, 2010; Recanzone and Cohen, 2010; Hackett, 2011; Hickok and Saberi, 2012). In these models, a posterior (dorsal) stream via posterior temporal lobe to inferior parietal cortex is involved in the processing of spatial aspects of sounds or information needed in auditory–motor integration (‘where’ or ‘how’ pathway), and an anterior (ventral) pathway through anterior temporal areas to inferior frontal cortex, in turn, is involved in the analysis of auditory objects and meaning of speech (‘what’ pathway). As pitch is an important auditory identity cue, such models predict that auditory cortical areas differ in their sensitivity to pitch and location. This idea is supported by the results of a large number of human neuroimaging studies (for reviews see, Arnott et al., 2004; Ahveninen et al., 2014; Alho et al., 2014; Lee et al., 2014). However, some previous studies have reported activations associated with spatial tasks in areas of the anterior superior temporal gyrus (STG; i.e., within the putative ‘what’ pathway) and activations associated with pitch tasks in posterior STG and in the inferior parietal lobule (IPL; i.e., within the putative ‘where’ pathway; Griffiths and Warren, 2002; Arnott et al., 2004; Hall and Plack, 2009; Rinne et al., 2009, 2012; Hill and Miller, 2010; Schadwinkel and Gutschalk, 2010). Further, although most current models of AC operations emphasize feature-specific analysis of auditory information, it is clear that AC responses are not a fixed function of the acoustic stimulus properties and that active listening tasks strongly modulate auditory processing (Petkov et al., 2004; Rinne et al., 2005; Weinberger, 2012; Bizley and Cohen, 2013; Yin et al., 2014). Our previous studies have shown that activations in AC depend on the task so that activations in areas of anterior STG are enhanced during discrimination but not during n-back memory task, whereas activations in posterior STG and IPL are enhanced during n-back but not during discrimination. This anterior–posterior distinction is detected quite similarly when analogous discrimination and n-back tasks are performed on pitch-varying sounds (Rinne et al., 2009), location-varying sounds (Rinne et al., 2012, 2014), or vowels (Harinen and Rinne, 2014). Thus, it is evident that the main features of these activation patterns are due to characteristics and requirements of the discrimination and n-back tasks and not due to stimulus-dependent processing of pitch, location, or vowel information as such. However, these studies did not directly investigate the similarities and differences between stimulus-dependent and task-dependent activations during pitch and location processing. Therefore, in the present functional magnetic resonance imaging (fMRI) study, we compared activations in conditions in which the pitch and location of the sounds as well as the task were systematically varied.

Our subjects were presented with sound pairs in which both pitch and location varied, only one feature varied (e.g., pitch) and the other feature (e.g., location) was diffuse (not salient), or both features were diffuse. In different blocks, subjects performed visual, auditory discrimination, and auditory n-back memory tasks. During visual tasks, subjects detected changes in orientation or spatial frequency in an independent sequence of Gabor gratings presented concurrently with the sounds. During auditory discrimination tasks, subjects were to detect pitch or location differences within the sound pairs. During auditory n-back memory tasks, subjects indicated when a sound pair belonged to the same pitch (low, medium, or high) or location (left, middle, or right) category as the one presented one or two trials before. Activations to sounds during visual tasks were analyzed to investigate stimulus-dependent processing of pitch and location in the absence of directed auditory attention, whereas the auditory task conditions allowed us to examine both stimulus-dependent and task-dependent effects.

In particular, we tested the following hypotheses. First, according to the current auditory models, stimulus-dependent processing of pitch and location should be associated with enhanced activations in distinct areas of anterior and posterior STG, respectively. This hypothesis was first tested on the basis of activations to pitch-varying and location-varying sounds presented during the visual task (i.e., no directed auditory attention). Further, based on the results of our previous studies comparing activations during discrimination and n-back memory tasks performed on either pitch-varying (Rinne et al., 2009) or location-varying (Rinne et al., 2012) sounds, we hypothesized that the task-dependent activation patterns associated with these tasks should be similar irrespective of whether the tasks are performed on sounds in which either or both features vary. However, if pitch and location are processed in separate pathways, then the distinction between pitch and location processing should also be present during auditory tasks. This idea was tested by comparing activations to identical sounds presented during pitch and location tasks and by comparing activations associated with task-irrelevant pitch and location variation during auditory tasks. Finally, as it is known that auditory attention enhances the representation of task-relevant feature information (e.g., Alain et al., 2001; Lee and Middlebrooks, 2010; Da Costa et al., 2013; Yin et al., 2014), we hypothesized that pitch and location tasks should enhance activations specifically in those areas that also show activation enhancements associated with stimulus-dependent pitch and location processing, respectively.

## Materials and Methods

### Subjects

Subjects (*N* = 22, 14 men) were 18–36 years of age (mean 25.5 years), right-handed, had normal hearing, normal or corrected-to-normal vision, and no history of psychiatric or neurological illnesses (all data based on subjects’ own report). Each subject signed an informed written consent before taking part in the experiment. The study protocol was approved by the research ethics committee of the Faculty of Behavioral Sciences, University of Helsinki, Finland.

### Stimuli and Tasks

Iterated rippled noise (IRN) bursts with a salient pitch were generated by iteratively adding delayed Gaussian noise (five iterations, delays 0.7–5 ms corresponding to pitch range 200–1400 Hz, 200 equal mel steps). In order to create sounds with a diffuse pitch, these IRN bursts were then demodulated (Barker et al., 2012) and mixed with white noise (ratio 3:7). Duration of sounds was set to 90 ms including 30-ms raised-cosine onset and offset ramps. All sounds were convolved with head-related transfer functions of a dummy head (MIT Media Lab)^1^ to create 25 distinct virtual spatial locations between ±120° (step 10°) in azimuth. To create sounds with a diffuse location, left and right channels were decorrelated using the Gram–Schmidt procedure (ρ = 0; locations -40–40°; e.g., Culling et al., 2001). Loudness was equalized by three experienced listeners and random intensity variation (0–10%) was added to mask any remaining systematic loudness differences.

The stimuli were presented in 12.7-s task blocks alternating with 7-s breaks with no stimuli. During task blocks, subjects were presented with concurrent but asynchronous streams of sound pairs (two 90-ms parts separated by a 20-ms gap; pair onset-to-onset interval 800–1000 ms, rectangular distribution, step 10 ms) and visual Gabor gratings (duration 100 ms; onset-to-onset interval 240–320 ms, rectangular distribution, step 20 ms). During the breaks, subjects focused on a fixation cross (black on gray background) presented in the middle of the screen. A task instruction symbol appeared on the screen 5 s prior to the next block and was presented until the end of the block.

During each block, either both pitch and location were salient (P1L1), one feature was salient and the other diffuse (P1L0 or P0L1), or both features were diffuse (P0L0; see **Table 1**). In tasks performed on P1L1 sounds, the task-irrelevant feature (i.e., location during pitch tasks or pitch during location tasks) did not vary within the sound pair. In the pitch discrimination tasks, subjects were required to indicate when the two parts of a sound pair were different in pitch by pressing a button with their right hand index finger. Similarly, in the location discrimination tasks, subjects indicated when the parts of a sound pair were different in location. The within-pair pitch and location differences were adjusted individually for each subject during pre-fMRI training according to their pitch and location discrimination sensitivity, respectively. In pitch tasks, the within-pair pitch difference was 3–10 steps (corresponding to 12–88 Hz). In location tasks, the location difference was 2–5 steps (20–50°) with an extra step (10°) added to sounds at ±40–120°. In discrimination P0L0 tasks the two parts of a sound pair were always identical, i.e., there were no targets. The P0L0 tasks were included to investigate task-dependent activations associated with pitch and location discrimination tasks in the absence of stimulus-dependent processing of the task-relevant feature (i.e., because of this, there were no targets in the P0L0 tasks). In pitch and location n-back tasks, subjects were required to indicate when a sound pair belonged to the same pitch or location category, respectively, as the one presented either one (1-back) or two (2-back) trials before. In n-back tasks, the two parts of a sound pair were equal in pitch and location (i.e., no within-pair variation). There were three pitch and location categories: low (corresponding to 200–318 Hz), medium (587–762 Hz), high (1148–1400 Hz), left (80–120° to the left of midline), middle (locations between ±20° of midline) and right (80–120° to the right of midline). The pitch and location sequences were balanced so that in each block with location-varying sounds there was a similar amount of sounds presented from the left, right, and center locations and in each block with pitch-varying sounds there was a similar amount of low, middle, and high pitch sounds.

**Table 1:** Experimental conditions.

Task	Task-relevant feature	Sounds
Discrimination	Pitch	P1L1
		P1L0
		P0L0
	Location	P1L1
		P0L1
		P0L0
1-back	Pitch	P1L1
		P1L0
	Location	P1L1
		P0L1
2-back	Pitch	P1L1
		P1L0
	Location	P1L1
		P0L1
Visual	Gabor orientation/frequency	P1L1
		P1L0
		P0L1
		P0L0


*Sounds varied both in pitch and spatial location, only in one feature, or both features were diffuse. P1/P0 salient/diffuse pitch. L1/L0 salient/diffuse location.*

The visual stimuli consisted of Gabor gratings (4° subtended angle). Gabor orientation (0–180°) and spatial frequency (0.4–1 cpd) changed 2–3 times in each block. In visual tasks, subjects were required to detect the changes in orientation (20°; 50% of blocks) or spatial frequency (0.2 cpd) of the Gabor gratings. During visual task blocks, sound sequences used in pitch and location discrimination tasks (no targets) were presented. The visual stimuli were similarly presented during visual and auditory tasks.

The auditory stimuli were delivered using a KAR ADU2a audio system (Unides Design, Helsinki, Finland) via plastic tubes through a porous EAR-tip (ER3, Etymotic research, Elk Grove Village, IL, USA). Scanner noise (ca. 97 dB, A-weighted measurement inside the head coil) was attenuated by the use of EAR-tips and ear muffs as well as viscous foam pads attached to the sides of the head coil. The visual stimuli and the task instruction symbols were presented in the middle of a screen viewed through a mirror attached to the head coil. The task instruction symbols consisted of two vertical arrowheads (∨) pointing away from each other, both pointing upward, or pointing toward each other for discrimination, n-back and visual tasks, respectively. Pitch tasks were indicated by red and location tasks by blue arrowheads. For the discrimination P0L0 and 2-back tasks both arrowheads were red/blue (i.e., indicating a demanding task), whereas for the other discrimination tasks and 1-back tasks the upper sign was red/blue and the lower white. In visual tasks, the frequency detection task was indicated by two black arrowheads, and, for the orientation detection task, the other arrowhead was black and the other white.

The experiment was conducted in two parts separated by a brief pause. During the pause subjects were instructed to remain silent and still. In both parts, each of the 18 conditions (**Table 1**) was repeated five times in random order so that in total (2 × 5 × 18) 180 blocks were presented during the experiment. There were 2–3 targets in each auditory and visual block, except for the discrimination P0L0 task which contained no targets (the first and last part of the sound were always the same). The experiment was controlled using the Presentation software (version 14.9, Neurobehavioral Systems, Albany, CA, USA).

### Pre-fMRI Training

Before fMRI, each subject was carefully trained (2 h of training in two sessions, second one within a week before scanning) to perform the tasks. During the pre-fMRI training, subjects were informed that the tasks were intentionally very demanding and that there were few or no targets in each block. During training, the pitch/location discrimination P0L0 tasks contained pitch/location targets.

### Analysis of the Behavioral Data

Mean hit rates (HRs), false alarm rates (FaRs), and reaction times (RTs) were calculated separately for each task. Responses occurring between 200 and 1300 ms from target onset were accepted as hits. Other responses (i.e., extra responses after a hit or responses outside the response window) were considered as false alarms. HR was defined as the number of hits divided by the number of targets. FaR was defined as the number of false alarms divided by the number of non-targets. Mean RT was calculated only for hits. HRs and FaRs were used to compute the *d*′ [*d*′ = Z(HR) – Z(FaR)]. Performance (*d*′) was analyzed using repeated-measures ANOVAs and *t*-tests.

### fMRI Data Acquisition and Analysis

**FIGURE 1:**
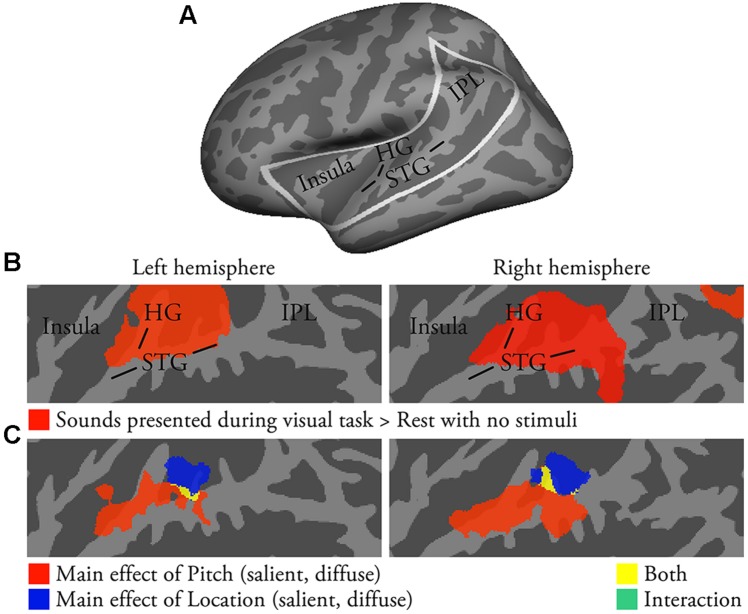
**Activations to pitch-varying and location-varying sounds presented during visual tasks (i.e., no directed auditory attention; *N* = 22; *p* < 0.05, cluster-corrected *Z* > 2.3).** **(A)** Inflated left-hemisphere cortical surface (light gray, gyri; dark gray, sulci). Areas of auditory cortex, insula and IPL included in our analyses are outlined in white. Results in **(B,C)** and in **Figures 2–4** are shown on flattened two-dimensional maps of these areas. Flat-Mapper tool (www.ebire.org/hcnlab/cortical-mapping) can be used to compare the flattened 2D maps to 3D anatomy. **(B)** Areas in red showed enhanced activations to pitch-varying and location-varying sounds presented during visual tasks (vs. rest). STG superior temporal gyrus, HG Heschl’s gyrus, IPL inferior parietal lobule. **(C)** The results of an ANOVA with factors Pitch (salient, diffuse) and Location (salient, diffuse).

Functional magnetic resonance imaging data were acquired with a 3 T MAGNETOM Skyra scanner (Siemens Healthcare, Erlangen, Germany) using a standard 20-channel head-neck coil. First, high-resolution anatomical image (sagittal slices, slice thickness 1.0 mm, in-plane resolution 1.0 mm × 1.0 mm) was acquired. Second, functional images (GE-EPI; TR 2070 ms, TE 30 ms, flip angle 78°, voxel matrix 96 × 96, FOV 18.9 cm × 18.9 cm, slice thickness 2.0 mm with no gap, in-plane resolution 2.0 mm × 2.0 mm, 27 slices) were acquired in two 30 min runs with a short break in between. The middle EPI slices were aligned along the Sylvian fissures based on the anatomical image. The imaged area covered the superior temporal lobe, insula, and most of the inferior parietal lobe in both hemispheres (**Figure 1A**). Finally, a T2-weighted image using the same imaging slices but with denser in-plane resolution was acquired (TR 4500, TE 100 ms, voxel matrix 256 × 240, FOV 24 cm, slice thickness 2.0 mm).

FreeSurfer was used for reconstruction of cortical surfaces and coregistration (version 5.3.0^2^). Functional data were motion-corrected, resampled to the standard cortical surface, and surface-smoothed (5 mm FWHM). Global vertex-wise analysis was performed in surface space using FILM (FMRIB’s Improved Linear Model; FSL version 5.0.7^3^). Each task (18) was included as a separate explanatory variable, and the hemodynamic response function was modeled with a gamma function (mean lag 6 s, SD 3 s) and its temporal derivative. A second level analysis using fixed effects combined the data from the two runs. Third level group analysis was performed using FSL’s PALM (Permutation Analysis of Linear Models; version alpha26, Winkler et al., 2014). Parameter estimates (from second level analysis) were compared using repeated-measures ANOVAs and *t*-tests. Significance was assessed by permutation inference (10000 permutations). Correction for multiple comparisons was performed using cluster-mass correction (cluster forming threshold *Z* > 2.3). For visualization, results were converted to 2D using Mollweide projection (Python libraries matplotlib and basemap^4^).

### Region of Interest (ROI) Analysis

A *post hoc* analysis was conducted to investigate IPL activations in more detail. First, rectangular ROIs were defined in the flattened 2D space to cover IPL in both hemispheres (**Figure 4D**). That is, the ROIs were anatomically defined independent of activation. Then, percent signal changes relative to rest (the 7-s between-task rest blocks with no stimuli) were calculated for each subject and condition and compared (10000 permutations).

## Results

### Task Performance

Subjects successfully performed the demanding tasks during fMRI (**Table 2**). Across all auditory tasks, excluding discrimination tasks with diffuse pitch and location (P0L0), mean *d*′ was 2.0. In visual tasks, mean *d*′ was 3.3. Mean RTs in auditory and visual tasks were 772 and 894 ms, respectively. In the pitch and location discrimination P0L0 tasks there were no targets as both features were diffuse. Yet, subjects gave target responses (for responses per block, see **Table 2**) also in these conditions suggesting that they tried to perform the tasks as instructed.

**Table 2:** Task performance in auditory tasks.

		Mean *d*′ (SEM)		Mean responses per block (SEM)	
			
Auditory task	Sounds	Pitch task	Location task	Pitch task	Location task
Discrimination	P1L1	2.0 (0.1)	2.0 (0.1)	27 (2)	21 (2)
	P1L0/P0L1	1.8 (0.1)	2.1 (0.1)	27 (3)	26 (2)
	P0L0	–	–	15 (2)	13 (2)
1-back memory	P1L1	1.9 (0.1)	2.5 (0.2)	26 (2)	22 (1)
	P1L0/P0L1	2.2 (0.2)	2.8 (0.2)	28 (2)	26 (1)
2-back memory	P1L1	1.2 (0.1)	1.8 (0.1)	20 (2)	19 (1)
	P1L0/P0L1	1.5 (0.1)	2.1 (0.2)	23 (2)	23 (1)

		**Mean hit rate (SEM)**		**Mean false alarm rate (SEM)**	
			
**Auditory task**	**Sounds**	**Pitch task**	**Location task**	**Pitch task**	**Location task**

Discrimination	P1L1	65 (4.7)	60 (3.9)	8.3 (1.0)	5.0 (0.9)
	P1L0/P0L1	62 (4.6)	68 (3.9)	9.3 (1.4)	7.4 (1.2)
1-back memory	P1L1	64 (4.1)	70 (4.5)	8.2 (1.3)	4.1 (0.7)
	P1L0/P0L1	72 (4.3)	81 (3.6)	8.3 (1.3)	4.7 (0.7)
2-back memory	P1L1	39 (4.1)	54 (3.9)	8.1 (1.3)	4.9 (0.8)
	P1L0/P0L1	50 (4.0)	63 (4.4)	8.1 (1.4)	6.0 (1.0)


Performance (*d*′) in pitch and location P1L1 tasks was examined by ANOVA with factors Task (discrimination, 2-back) and Task-Relevant Feature (pitch, location). The ANOVA showed significant main effects (Task, *F*_1,21_ = 11.4, *p* < 0.01; Task-Relevant Feature, *F*_1,21_ = 10.9, *p* < 0.01) and an interaction (Task × Task-Relevant Feature, *F*_1,21_ = 7.9, *p* < 0.05). Performance was lower in pitch than location 2-back P1L1 task (*t*_21_ = 4.0, *p* < 0.01). This difference was not significant in discrimination tasks (*t*_21_ = 0.7, *p* > 0.4).

Effects of task-irrelevant pitch and location variation on performance were investigated using separate ANOVAs with factors Task (discrimination, 2-back) and Task-Irrelevant Pitch/Location (salient, diffuse). Performance in pitch tasks was not affected by task-irrelevant location (main effect of Task-Irrelevant Location, *F*_1,21_ = 0.1, *p* > 0.7), but in location tasks performance was lower when task-irrelevant pitch was present (main effect of Task-Irrelevant Pitch, *F*_1,21_ = 5.8, *p* < 0.05). Interactions were not significant (in both ANOVAs, *p* > 0.05). Performance in visual tasks was not different in blocks with P1L1, P1L0, P0L1, and P0L0 sounds (*F*_3,21_ < 1.4, *p* > 0.2).

### Activations to Pitch-Varying and Location-Varying Sounds during Visual Task

Activations to task-irrelevant sounds presented during the visual task were analyzed to investigate stimulus-dependent auditory processing in the absence of directed auditory attention or task. Activations were stronger during visual task blocks than during the 7-s between-task rest periods with no stimuli in areas extending from HG to posterior STG (**Figure 1B**; a similar extent of activations was detected using a threshold of uncorrected *p* < 0.1). Visual task activations were then entered into an ANOVA with factors Pitch (salient, diffuse) and Location (salient, diffuse). The ANOVA revealed a significant main effect of Pitch in anterior–middle STG and lateral HG (**Figure 1C**, red and yellow) and a main effect of Location in middle–posterior STG and planum temporale (PT; blue and yellow). Pitch and location main effects overlapped in anterolateral PT (yellow). Direct contrasts investigating these main effects indicated that pitch variation was associated with enhanced activations in anterior–middle STG, whereas location variation was associated with enhanced activations in anterior PT (not shown).

### Activations to Pitch-Varying and Location-Varying Sounds during Auditory Tasks

In pitch discrimination and pitch n-back memory tasks, the pitch-varying sounds either had salient or diffuse location (L1 or L0, respectively). Similarly, during location tasks, pitch was salient (P1) or diffuse (P0). This allowed us to investigate the effects associated with the processing of task-irrelevant and task-relevant pitch and location during auditory tasks.

**FIGURE 2:**
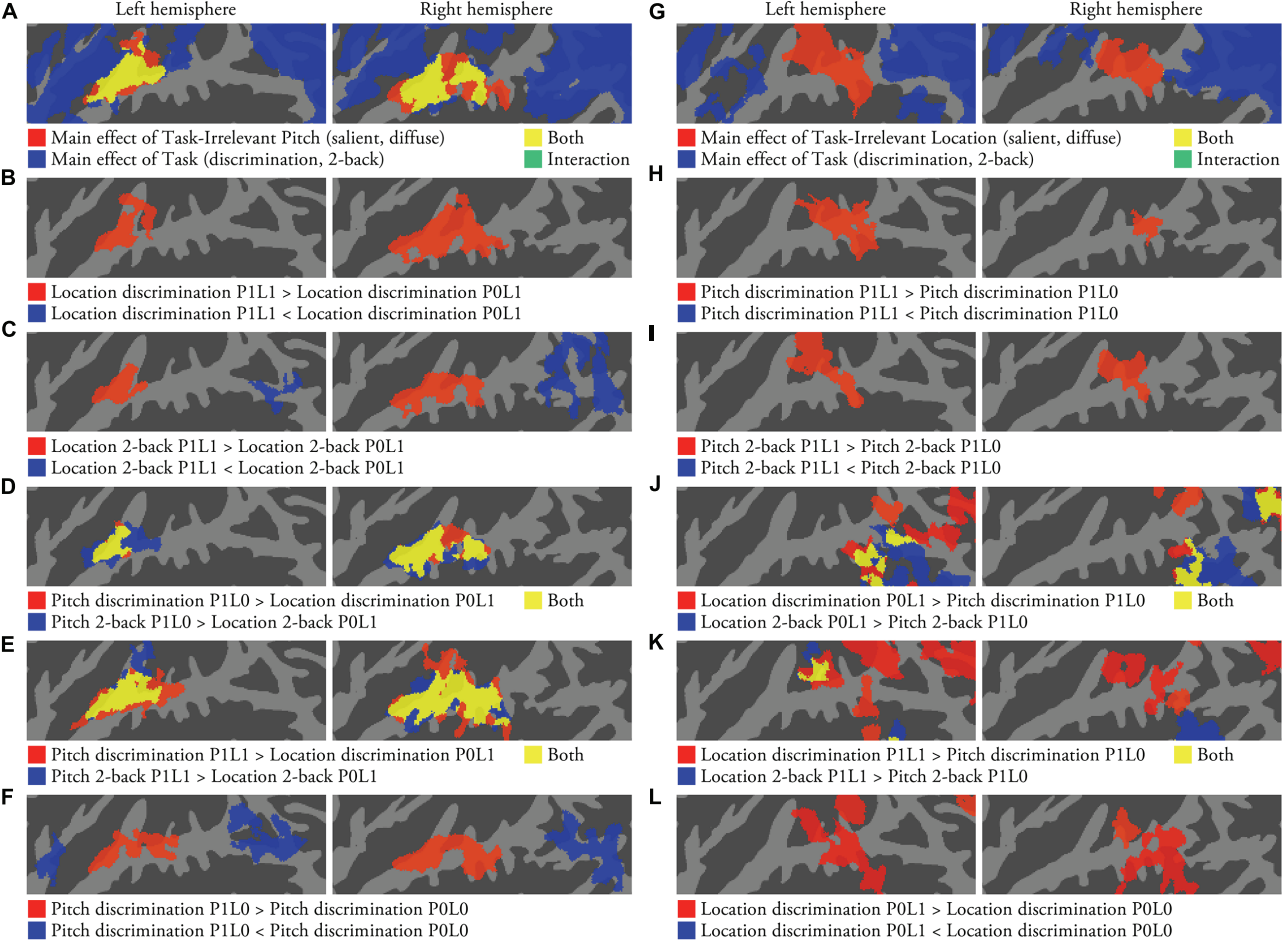
**Activations to pitch and location during auditory tasks (*N* = 22; *p* < 0.05, cluster-corrected *Z* > 2.3).** **(A)** The results of an ANOVA with factors Task-Irrelevant Pitch (salient, diffuse) and Task (discrimination, 2-back). **(B)** Areas showing pitch sensitivity during location discrimination task. **(C)** Areas showing pitch sensitivity during location 2-back task. **(D)** Areas where activations were stronger during pitch P1L0 than location P0L1 tasks. **(E)** Areas where activations were stronger during pitch P1L1 than location P0L1 tasks. **(F)** Comparison of activations during pitch discrimination P1L0 and P0L0 tasks. **(G–L)** The corresponding comparisons for location.

Activations to task-irrelevant pitch during location tasks were studied using an ANOVA with factors Task-Irrelevant Pitch (P1 salient, P0 diffuse) and Task (discrimination, 2-back). The ANOVA revealed a significant main effect of Task-Irrelevant Pitch in similar areas of STG that also were sensitive to presentation of sounds during visual task (**Figure 2A**, red and yellow). Main effect of Task was detected in wide areas extending from anterior insula to STG and IPL (blue and yellow). Direct contrasts showed that the processing of task-irrelevant pitch variation during location tasks was associated with enhanced activations in anterior STG (**Figures 2B,C**, red) and decreased activations in IPL, particularly during the 2-back task (**Figure 2C**, blue). At a more lenient threshold (uncorrected *p* < 0.05, not shown), some scattered areas in IPL showed activation decrements also during location discrimination tasks (location discrimination P1L1 < P0L1). Contrasts pitch P1L0/P1L1 vs. location P0L1 tasks (**Figures 2D,E**) and salient vs. diffuse-pitch tasks (P1L0 > P0L0; **Figure 2F**, red) showed similar enhanced activations in STG associated with the processing of task-relevant pitch. In addition, IPL activations were lower when the pitch discrimination task was performed on sounds with a salient pitch than with diffuse pitch (**Figure 2F**, blue).

Corresponding analyses for activations related to the processing of task-irrelevant location during pitch tasks revealed a significant main effect of Task-Irrelevant Location in posterior STG and PT (**Figure 2G**, red and yellow), and a main effect of Task in wide areas of the insula, STG and IPL (blue and yellow). Direct contrasts showed that during pitch tasks, task-irrelevant location was associated with enhanced activations in posterior STG and PT (**Figures 2H,I**, red). Comparisons between location P0L1 and pitch P1L0 tasks revealed significant activation enhancements for location P0L1 tasks mainly in IPL (**Figure 2J**). However, when location P1L1 tasks (i.e., with task-irrelevant variation of salient pitch) and pitch P1L0 tasks were compared, activation enhancements associated with location P1L1 tasks were also observed in PT (**Figure 2K**). Contrasts between location discrimination task with and without salient location cues (P0L1 vs. P0L0) showed enhanced activations to location discrimination P0L1 task in PT and posterior STG (**Figure 2L**, red). At a more lenient threshold (uncorrected *p* < 0.05, not shown), these contrasts also revealed that activations in IPL were lower when the location discrimination task was performed on sounds with a salient location than with diffuse location. Similar stimulus-dependent activations to task-irrelevant pitch and location were also detected during 1-back tasks (not shown).

### Activation Differences between Pitch and Location Tasks

**FIGURE 3:**
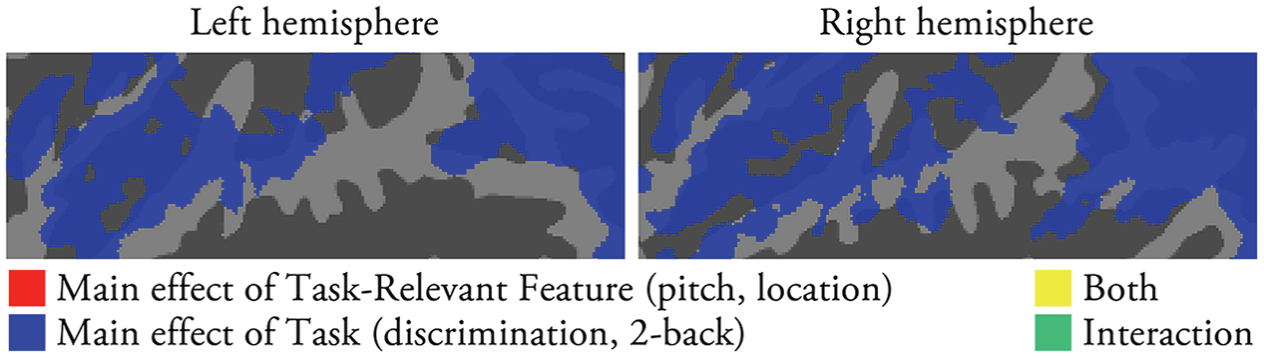
**Comparison of activations during auditory pitch and location tasks with the same sounds (*N* = 22; *p* < 0.05, cluster-corrected *Z* > 2.3).** The results of an ANOVA with factors Task-Relevant Feature (pitch, location) and Task (discrimination, 2-back) are shown.

The pitch and location tasks were also performed on identical P1L1 sounds. Activations during these tasks were compared using an ANOVA with factors Task-Relevant Feature (pitch, location) and Task (discrimination, 2-back). The ANOVA showed a significant main effect of Task in wide areas extending from anterior insula to posterior STG and IPL (**Figure 3**, blue). The main effect of Task-Relevant Feature and interaction were not significant. Correspondingly, the comparisons pitch vs. location 1-back P1L1 and pitch vs. location discrimination P0L0 tasks did not reveal significant activation differences (not shown).

### Task-Dependent Activations

**FIGURE 4:**
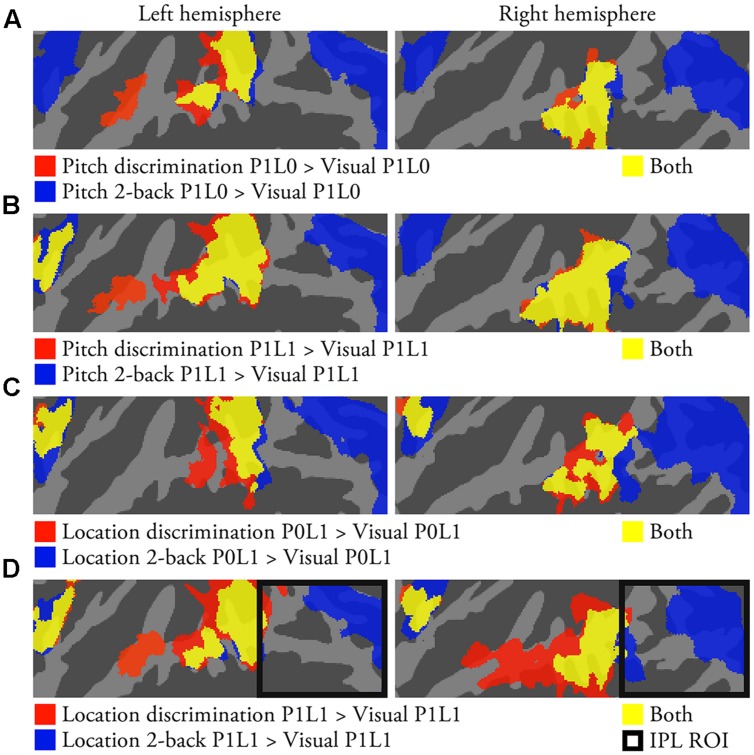
**Task-dependent activations (*N* = 22; *p* < 0.05, cluster-corrected *Z* > 2.3).** **(A)** Areas showing enhanced activations during pitch discrimination P1L0 or pitch 2-back P1L0 task as compared with visual task with same sounds. Corresponding comparisons for **(B)** pitch P1L1 tasks, **(C)** location P0L1 tasks, and **(D)** location P1L1 tasks. IPL ROIs are outlined in black **(D)**.

As compared with the visual task with identical sounds, discrimination tasks were associated with enhanced activations in anterior insula and anterior–posterior STG and 2-back tasks with enhanced activations in anterior insula, posterior STG, and IPL in all conditions (**Figures 4A–D**). Direct contrasts showed that discrimination tasks were associated with stronger activations in anterior–posterior STG, whereas 2-back tasks enhanced activations in insula and IPL. Further, activations in IPL increased and those in anterior STG decreased with increasing difficulty in n-back tasks (not shown).

### IPL ROI Analysis

**FIGURE 5:**
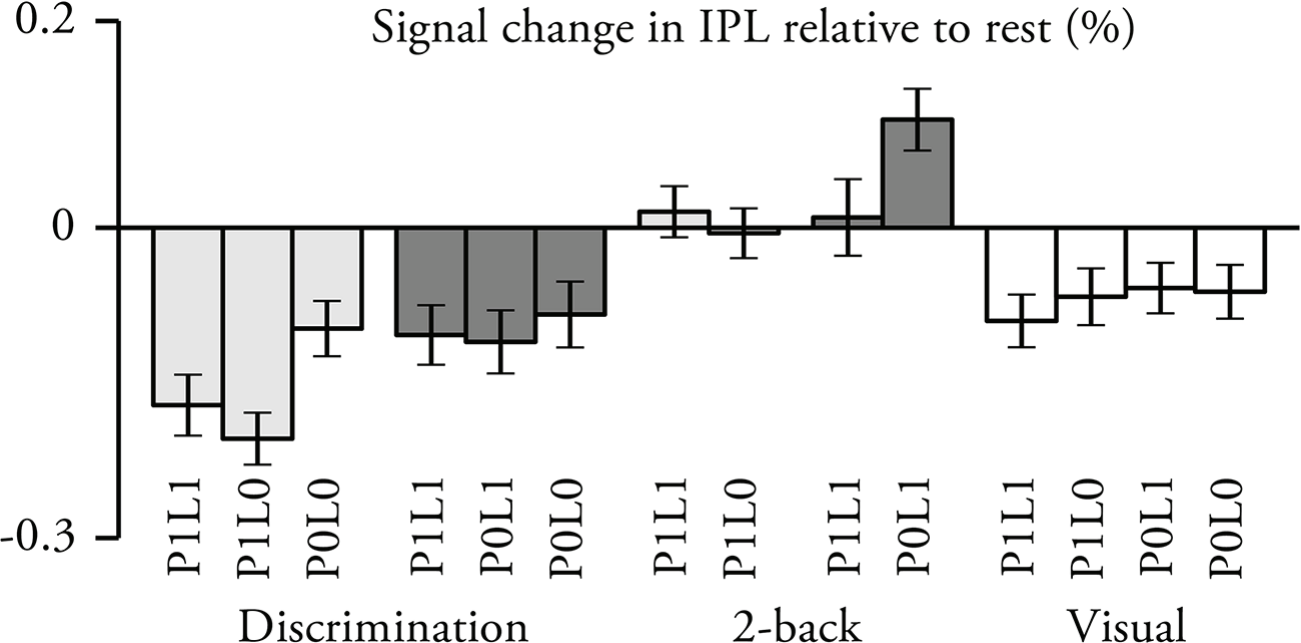
**Percent signal change in IPL relative to rest (*N* = 22; mean ± SEM; for ROI definition see **Figure 4D**).** Lighter gray, pitch tasks; darker gray, spatial tasks.

Vertex-wise analysis revealed some unexpected results in IPL. To investigate these results in more detail a *post hoc* ROI analysis was conducted (**Figure 5**). Activations in the IPL ROI were significantly lower than baseline (i.e., rest) during pitch and location discrimination and visual tasks (in all cases, *t*_21_ > 2.4, *p* < 0.05), and higher during location 2-back P0L1 task (*t*_21_ > 3.5, *p* < 0.01). As compared with the visual task with identical sounds, signal magnitudes were higher during all 2-back tasks (*t*_21_ > 1.8, *p* < 0.05) and lower during discrimination tasks except for location discrimination P1L1 (*t*_21_ > 1.7, *p* < 0.05; location discrimination P1L1, *t*_21_ = 0.5, *p* > 0.3). Signal magnitudes were higher during 2-back than during the corresponding discrimination tasks (all comparisons with identical sounds, *t*_21_ > 3.5, *p* < 0.01). Further, signal magnitudes were higher during location than pitch P1L1 tasks (*t*_21_ > 2.3, *p* < 0.05) but not different during pitch and location discrimination P0L0 tasks (*t*_21_ = 0.4, *p* > 0.3). Task-irrelevant pitch was associated with decreased signal magnitude during location 2-back (*t*_21_ = 2.3, *p* < 0.05) but not during location discrimination task (*t*_21_ = 0.3, *p* > 0.6). During visual tasks, signal magnitudes were not significantly modulated by the different stimulus conditions (ANOVA, *F*_3,21_ = 0.5, *p* > 0.6).

## Discussion

The present study aimed at better understanding the relationship between (obligatory) stimulus-dependent and task-dependent AC activations during pitch and location processing. In line with current theoretical models and the results of previous studies, we found areas of anterior–middle STG and PT to be sensitive to task-irrelevant pitch and location variation, respectively. However, when pitch and location were task-relevant this distinction was less clear. In particular, we found no enhanced activations in areas of PT that were specific to location tasks; instead, these areas also showed enhanced activations during pitch tasks. Consistent with previous studies, we observed enhanced IPL activations during active location processing. However, IPL did not show systematic sensitivity to task-irrelevant or task-relevant pitch or location.

### Task-Irrelevant and Task-Relevant Pitch and Location

We presented pitch-varying and location-varying sounds during a demanding visual task (i.e., no directed auditory attention) in order to investigate stimulus-dependent activations to these sounds. We found that task-irrelevant pitch and location were associated with distinct activations in anterior–middle STG and PT, respectively (**Figure 1C**), and that these activations were similar irrespective of whether one (e.g., pitch only, P1L0 vs. P0L0) or two features varied (e.g., pitch and location, P1L1 vs. P0L1). Thus, these results are consistent with the view that pitch and location are processed independently in separate areas of anterior and posterior AC (Romanski et al., 1999; Warren and Griffiths, 2003; Barrett and Hall, 2006; Rauschecker and Scott, 2009; Ahveninen et al., 2014; Alho et al., 2014).

If these activation differences observed during visual task arise due to obligatory processing of pitch and location information in separate brain areas, then similar activation differences should also be present during auditory tasks. Consistently, we found that task-irrelevant pitch (**Figures 2A–C**) and location (**Figures 2G–I**) during auditory tasks were associated with enhanced activations in rather similar areas that were also sensitive to these features during visual tasks. Further, the same areas that showed sensitivity to task-irrelevant pitch were also sensitive to task-relevant pitch irrespective of whether task-irrelevant location variation was present or not (**Figures 2D–F**). However, we found that activations in PT and IPL could not be fully predicted on the basis of obligatory processing of pitch and location. Activations in PT and IPL will be discussed in more detail below (PT activations and IPL activations).

### Pitch and Location Tasks with Identical Sounds

Previous studies have shown that auditory selective attention enhances the representation of task-relevant feature information (e.g., Alain et al., 2001; Lee and Middlebrooks, 2010; Da Costa et al., 2013; Yin et al., 2014). In the present study, we hypothesized that pitch and location tasks should be associated with enhanced activations specifically in the areas that show sensitivity to these features. However, we found no significant activation differences (in STG and PT) between pitch and location tasks performed on identical sounds (**Figure 3**). This suggests that enhanced stimulus-specific activations do not strongly contribute to the activation patterns observed during the present pitch and location tasks. It should be noted that in the present study we measured activations to broadband sounds with varying pitch and location. Evidently, such design is not optimal for detecting the effect of selective attention on the representation of a task-relevant frequency (e.g., Da Costa et al., 2013; Yin et al., 2014) or location. Nevertheless, the present results extend previous studies by showing that activations during pitch and location tasks are mainly due to (obligatory) stimulus-specific and task-dependent activations, while the contribution of attention-enhanced stimulus-specific activations (to pitch and location) is likely to be relatively small.

### Task-Dependent Activations

As compared with activations during visual tasks with identical sounds, discrimination tasks enhanced activations in anterior insula and anterior–posterior STG, whereas 2-back tasks were associated with enhanced activations in anterior insula, posterior STG and IPL (**Figure 4**). This anterior–posterior distinction between discrimination and n-back tasks replicates our previous results obtained in two separate studies using similar pitch (Rinne et al., 2009) and location tasks (Rinne et al., 2012; see also, Huang et al., 2013). Importantly, the present results indicate that the activation differences between discrimination and n-back tasks are quite similar irrespective of whether the tasks are performed on sounds that vary in pitch, location, or both features. Further, these robust task-dependent activation patterns are clearly distinct from the effects associated with pitch and location as discussed above. Note that, as compared to the visual task, IPL activations were enhanced during both pitch and location n-back tasks but not during discrimination tasks (**Figures 4** and **5**). These results provide further evidence that IPL activations cannot be explained by sensitivity to location but that its activations depend strongly on the requirements of the task. (For a more detailed discussion on the functional significance of task-dependent activations during discrimination and n-back tasks see, Rinne et al., 2009, 2012, 2014; Harinen and Rinne, 2014).

Although behavioral data suggested that the 2-back tasks were more difficult than the discrimination tasks, the activation differences between these tasks cannot easily be explained based on behavioral task difficulty alone. First, the activation patterns during discrimination and 2-back tasks appeared to be quite similar irrespective of behavioral performance. In particular, despite the fact that the largest performance differences were observed between pitch and location 2-back, no significant activation differences were observed between these tasks. Second, in our previous studies increasing task difficulty (as indicated by decreasing task performance) in pitch (Rinne et al., 2009) and location (Rinne et al., 2012) discrimination tasks was not associated with strong activation modulations. Together, these results strongly suggest that the present activation differences between discrimination and 2-back tasks are due to specific requirements of these tasks and not due task difficulty as such (see also, Harinen and Rinne, 2014; Rinne et al., 2014).

### PT Activations

As discussed above, PT showed significantly stronger activations to sounds with distinct location (L1) than to sounds with diffuse location (L0). This is consistent with the results of previous studies implicating PT in spatial processing (e.g., Warren and Griffiths, 2003; Barrett and Hall, 2006; van der Zwaag et al., 2011; Ahveninen et al., 2014; Shrem and Deouell, 2014). However, our results also show that PT activations cannot be fully predicted based on stimulus-dependent activations to location. Despite the distinct stimulus-level difference, no significant activations in PT to location were observed in the contrasts between location P0L1 and pitch P1L0 tasks featuring diffuse features on the opposite dimension (**Figure 2J**). This suggests that, in addition to sensitivity to location, PT activations were also increased during pitch tasks. Consistently, PT activations to location were observed in the contrast between location P1L1 and pitch P1L0 tasks (i.e., salient pitch in both; **Figure 2K**). Further, PT activations were stronger during auditory than visual tasks and during discrimination than n-back tasks, but PT activations were not different during location and pitch tasks performed on identical sounds with both salient features (P1L1). These results suggest that although PT is sensitive to location during all task conditions, it also shows sensitivity to pitch and to the requirements of the auditory tasks. The present results are consistent with the idea that PT is not a dedicated spatial processing area but that PT might act as a more general ‘computational hub’ engaged in analysis and segregation of sound patterns, matching incoming and previously stored patterns, and gating information for other cortical areas for further processing (Griffiths and Warren, 2002; Bizley and Cohen, 2013). Further, PT might show location sensitivity because it uses spatial information for auditory source separation rather than for spatial processing *per se* (Smith et al., 2010). The present enhanced PT activations during discrimination tasks are in line with this idea, as the discrimination tasks required detailed analysis of each sound pair, whereas in the n-back task the within-pair differences were not relevant (i.e., the demands for source segregation were probably lower in the n-back task). Finally, the present results are consistent with the view that spatial-related functions involve anterior areas of PT (activations to location extended to posterior HG, **Figure 1C**), whereas the more posterior areas of PT are involved in task-related operations (task-related activation enhancements were focused on the posterior parts of the supratemporal plane, **Figure 4**; Hickok and Saberi, 2012).

### IPL Activations

In the present study, activations in IPL were stronger during location P0L1 than pitch P1L0 tasks (**Figure 2J**). However, when the stimuli were presented during a visual task designed to direct attention away from the sounds, activations to pitch and location were detected in anterolateral STG and PT, respectively, but not in IPL (**Figure 1C**). Similarly, during pitch tasks, activations to task-irrelevant location were detected in PT, but not in IPL (**Figures 2G–I**). Together these results seem to suggest that IPL is sensitive to location during active location tasks (e.g., Alain et al., 2001, 2010; Zatorre et al., 2002; Zimmer and Macaluso, 2005; Rämä, 2008; Paltoglou et al., 2011). Interestingly, we also found that IPL activations decreased during location tasks when the sounds contained task-irrelevant pitch (**Figure 2C**) and during pitch discrimination when pitch was salient (i.e., pitch discrimination P1L0 vs. P0L0; **Figure 2F**). Further, our *post hoc* ROI analysis showed that IPL activations were lowest during pitch discrimination (P1L1 and P1L0) tasks (**Figure 5**). Based on these results, it could be speculated that during auditory tasks IPL is sensitive to pitch rather than to location so that IPL activations decrease during processing of both task-relevant and task-irrelevant pitch. Task-irrelevant pitch variation could also interfere with operations in IPL (e.g., associated with task-relevant location processing or location memory; Delogu et al., 2014; Joseph et al., 2015).

Taken together, the present results suggest that task-dependent activations in IPL are modulated by the characteristics of the stimuli (e.g., location 2-back P0L1 vs. P1L1) and task, but IPL shows no distinct stimulus-dependent sensitivity to pitch or location when attention is directed away from the sounds.

### Implications for Models of Human AC

**FIGURE 6:**
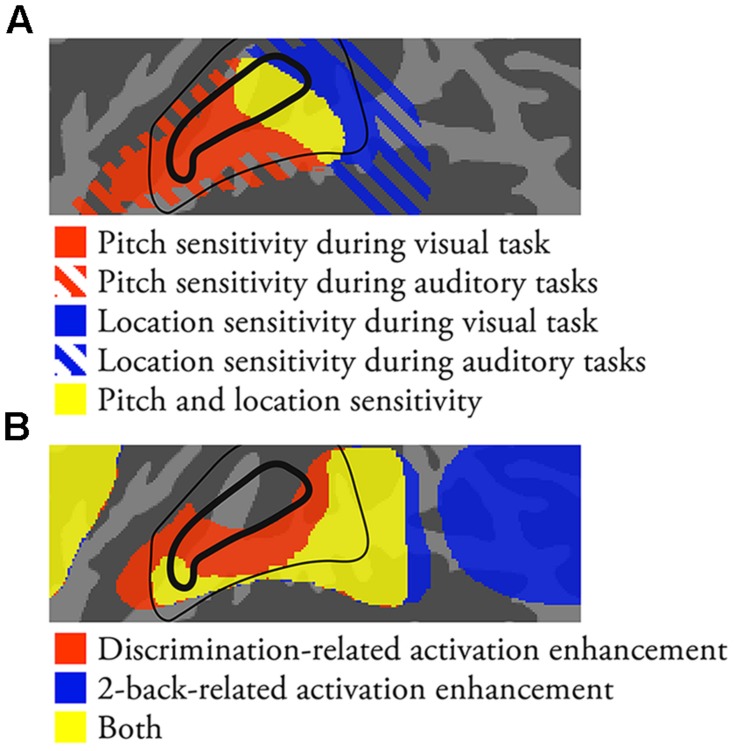
**A schematic summary of the present results together with a previously proposed model of human ACFs (Woods et al., 2010).** Core and belt ACFs are enclosed by a thick and thin black line, respectively. **(A)** Areas showing pitch and location sensitivity. **(B)** Areas showing task-dependent activations.

Parallel information processing streams are at the core of current theoretical models of human AC. Although the functional roles and organization of these streams are still debated, it is broadly accepted that pitch and location processing differently engage independent anterior and posterior streams, respectively. Based on studies in animals, it is also assumed that human AC consists of core, belt and parabelt areas that are further divided into several functional auditory cortical fields (ACFs; Kaas and Hackett, 2000; Recanzone and Cohen, 2010; Hackett, 2011). Structural imaging of myelination and functional imaging of tonotopic organization have been used to localize core ACFs in humans (for reviews see, Moerel et al., 2014; Saenz and Langers, 2014). While human belt and parabelt areas can be identified in postmortem anatomical studies (Sweet et al., 2005), there are currently no agreed-upon methods to localize human ACFs outside the core. Recent studies suggest that advanced anatomical and functional imaging and analysis methods will provide more accurate information on the organization of human AC (e.g., Barton et al., 2012; Moerel et al., 2012; Cha et al., 2014; De Martino et al., 2014). Some previous studies also suggest that human core, belt, and parabelt areas could be delineated based on their distinctive stimulus responses (e.g., pure tones vs. band-passed noise vs. conspecific vocalizations; Wessinger et al., 2001; Chevillet et al., 2011). We here propose several task-related distinctions between anterolateral STG, anterior PT, posterior PT/STG and IPL that could provide additional information to better understand the functional organization of human AC. A schematic summary of the present results together with a previously proposed model of human core and belt ACFs (Woods et al., 2010) is shown in **Figure 6**.

#### Anterolateral STG (**Figure 6A**, Red Areas)

The present comparisons between activations during visual task blocks with pitch-varying and location-varying sounds revealed pitch-sensitive areas in anterolateral STG (**Figure 1C**). These areas could correspond to the rostral core, belt, and parabelt ACFs from which the anterior ‘what’ stream originates (for a review see, Recanzone and Cohen, 2010). Most of these areas were also associated with enhanced activations during both pitch and location discrimination tasks. These results suggest that, in addition to being sensitive to pitch, anterolateral STG is activated during auditory tasks especially when the auditory task requires detailed analysis of the sounds.

#### Anterior PT (**Figure 6A**, Yellow Areas in or Near Core)

In contrast to anterolateral STG, anterior PT showed sensitivity to both pitch and location (overlap of pitch and location main effects in **Figures 1C** and **2A** vs. **2G**). Activations in anterior PT were also stronger during discrimination than 2-back tasks. Some previous studies suggest that the primary AC (core field A1) extends posteriorly from HG (Woods et al., 2010; Saenz and Langers, 2014). Thus, anterior PT could correspond to A1 or posterior belt (CM or CL).

#### Posterior PT/STG (**Figure 6A**, Blue Areas; **Figure 6B**, Yellow Areas Posterior to Core)

Areas in posterior PT/STG showed sensitivity to location (**Figures 1C** and **2G**). Activations in these areas were also enhanced during both discrimination and n-back tasks irrespective of the task-relevant feature (**Figure 4**). During 2-back tasks STG activation increases were focused on posterior PT/STG, whereas in discrimination tasks activation enhancements extended also to more anterior STG areas. Thus, posterior PT/STG could be involved in more general functions required in both of these demanding tasks. Posterior PT/STG could correspond to posterior belt (CM or CL) or parabelt ACFs.

#### IPL (**Figure 6B**, Blue Areas Posterior to STG)

Inferior parietal lobule is not generally considered a part of AC *per se*, and it has been implicated in many other functions not limited to auditory modality. However, IPL activations during auditory tasks seem to be strongly coupled with those in STG so that as activations in one area increase those in the other area decrease. First, in line with previous studies, we found that IPL activations increased and activations in anterior STG decreased with increasing difficulty in n-back tasks (Rinne et al., 2009, 2012; Huang et al., 2013). Second, while discrimination tasks were associated with increased STG activations, the present results suggest that these tasks also modulate IPL activations. Namely, IPL activations were lower when pitch and location discrimination tasks were performed on sounds with a salient task-relevant feature (i.e., P1L0 or P0L1) as compared to when these tasks were performed on P0L0 sounds (**Figure 2F**). Third, while the processing of task-relevant and task-irrelevant pitch was associated with increased STG activations, IPL activations during auditory tasks were decreased in the presence of pitch (see, IPL activations). These results suggest that areas in anterolateral STG and IPL activate and deactivate in a dynamically coupled manner. Therefore, it is important to consider AC operations and its functional organization within a wider cortical network including IPL.

In line with current auditory models, we found a clear distinction between anterolateral STG (within the putative anterior ‘what’ pathway) and PT (posterior ‘where’ pathway). In these areas, stimulus-dependent activations to pitch and location were not modulated by variation in the other feature, consistent with the view that processing in the two streams is largely independent. However, it has also been suggested that the auditory processing streams are task-defined rather than feature-defined (Hickok and Saberi, 2012). In keeping with this idea, the present comparisons between discrimination and n-back tasks revealed a task-dependent distinction between anterior/posterior STG and posterior STG/IPL, respectively. Interestingly, while the results of the present stimulus-dependent comparisons are consistent with the view that PT is functionally distinct from anterolateral STG, the task comparisons suggest that PT behaves in a similar manner as areas in anterior STG. In addition, we found that pitch and location tasks did not systematically enhance activations in those areas that showed sensitivity to these features. Taken together, the present results suggest that stimulus-dependent and task-dependent activations cannot be easily explained by a two-stream model in which the streams are either feature-defined or task-defined. Rather, the present results indicate that, although areas of AC show sensitivity to pitch and location, its activations to pitch-varying and location-varying sounds depend in a complex manner on the requirements of the tasks performed on these sounds. The present results also provide evidence for an important role of IPL during auditory tasks, although IPL is probably not implicated in stimulus-dependent processing of pitch and location as such.

## Conflict of Interest Statement

The authors declare that the research was conducted in the absence of any commercial or financial relationships that could be construed as a potential conflict of interest.

## Footnotes

1 http://sound.media.mit.edu/resources/KEMAR.html

2 www.freesurfer.net

3 www.fmrib.ox.ac.uk/fsl

4 http://matplotlib.sourceforge.net
